# Bilateral multiple evanescent white dot syndrome documented and followed by swept-source OCT angiography: a case report

**DOI:** 10.1186/s12348-025-00542-1

**Published:** 2025-11-03

**Authors:** Arsham Salameti, Jon Roger Eidet

**Affiliations:** 1 Faculty of Medicine, University of Oslo, Oslo, Norway; 2 Department of Ophthalmology, Oslo University Hospital, Oslo, Norway

**Keywords:** MEWDS, Bilateral, Swept-source OCTA, Choriocapillaritis, Primary inflammatory choriocapillaropathies, APMPPE

## Abstract

**Background:**

Bilateral Multiple Evanescent White Dot Syndrome (MEWDS) is rare and may mimic other Primary Inflammatory Choriocapillaropathies (PICCPs) such as Acute Posterior Multifocal Placoid Pigment Epitheliopathy (APMPPE). Swept-source optical coherence tomography angiography (SS-OCTA) offers improved visualization of the choriocapillaris and may facilitate early differential diagnosis.

**Case report:**

A healthy 32-year-old man presented with acute bilateral visual field disturbances and central vision loss in the left eye. Multimodal imaging, including SS-OCTA, demonstrated outer retinal layer attenuation and mild choriocapillaris flow deficit in the right eye, with no flow deficit in the left eye. The findings were more consistent with MEWDS than with APMPPE. Serial follow-up revealed spontaneous recovery of best-corrected visual acuity (BCVA) to 20/16 in both eyes, accompanied by normalization of all imaging parameters.

**Conclusion:**

This case highlights the diagnostic importance of multimodal imaging, particularly SS-OCTA, in atypical presentations of MEWDS. Early and accurate identification helps avoid unnecessary treatment and prompts appropriate investigation when more serious pathology is suspected.

## Introduction

Multiple Evanescent White Dot Syndrome (MEWDS) is a rare, self-limiting inflammatory choriocapillaropathy classified as a Primary Inflammatory Choriocapillaropathy (PICCP), a group that includes entities such as multifocal choroiditis with panuveitis, punctate inner choroiditis, and Acute Posterior Multifocal Placoid Pigment Epitheliopathy (APMPPE) [1–3]. Multiple Evanescent White Dot Syndrome typically affects young, otherwise healthy individuals, often women, and usually presents unilaterally with an acute onset of photopsia, scotomas, and reduced vision. Fundus examination reveals multiple, small, grey-white lesions at the level of the retinal pigment epithelium (RPE) and outer retina, often accompanied by a granular fovea [1, 3].

Although classically unilateral, bilateral cases have been described [4–6]. The disease is thought to result from transient inflammation of the choriocapillaris, causing secondary ischemic injury to the outer retina and photoreceptors [3]. Differential diagnosis with APMPPE is clinically relevant, as the latter is more often associated with systemic vasculitis and neurological complications [7].

Spectral-domain OCT (SD-OCT) is valuable for documenting outer retinal changes, but does not directly visualize the choriocapillaris perfusion. Swept-source OCTA (SS-OCTA) offers deeper penetration and enhanced imaging of the choriocapillaris, facilitating differential diagnosis in ambiguous cases [8–11].

This case demonstrates the use of SS-OCTA in a young man presenting with bilateral MEWDS. The bilateral manifestation posed a diagnostic challenge, where APMPPE was a clinically relevant consideration to rule out. Longitudinal follow-up with SS-OCTA provided valuable insights into choriocapillaris perfusion and structural recovery, highlighting both the potential and limitations of this modality in the diagnostic workup of Primary Inflammatory Choriocapillaropathies.

## Case report

A 32-year-old Pakistani man was referred to Oslo University Hospital from a private ophthalmologist due to a two-day history of bilateral visual disturbances, including decreased reading vision in the left eye and peripheral scotomas in both eyes. He reported a headache both six days before and on the day of presentation, but denied symptoms of a viral prodrome or any systemic symptoms. He had recently relocated to Norway and had no relevant medical history or current medications.

His best-corrected visual acuity (BCVA) was 20/16 in his right eye and 20/80 in his left eye. He had paracentral scotomas in his right eye. Ultra-widefield imaging (Optomap), fundus autofluorescence photography (FAF), SD-OCT, SS-OCTA, fluorescein angiography (FA), and indocyanine green angiography (ICGA) were performed at the first visit.

**Fig. 1:**
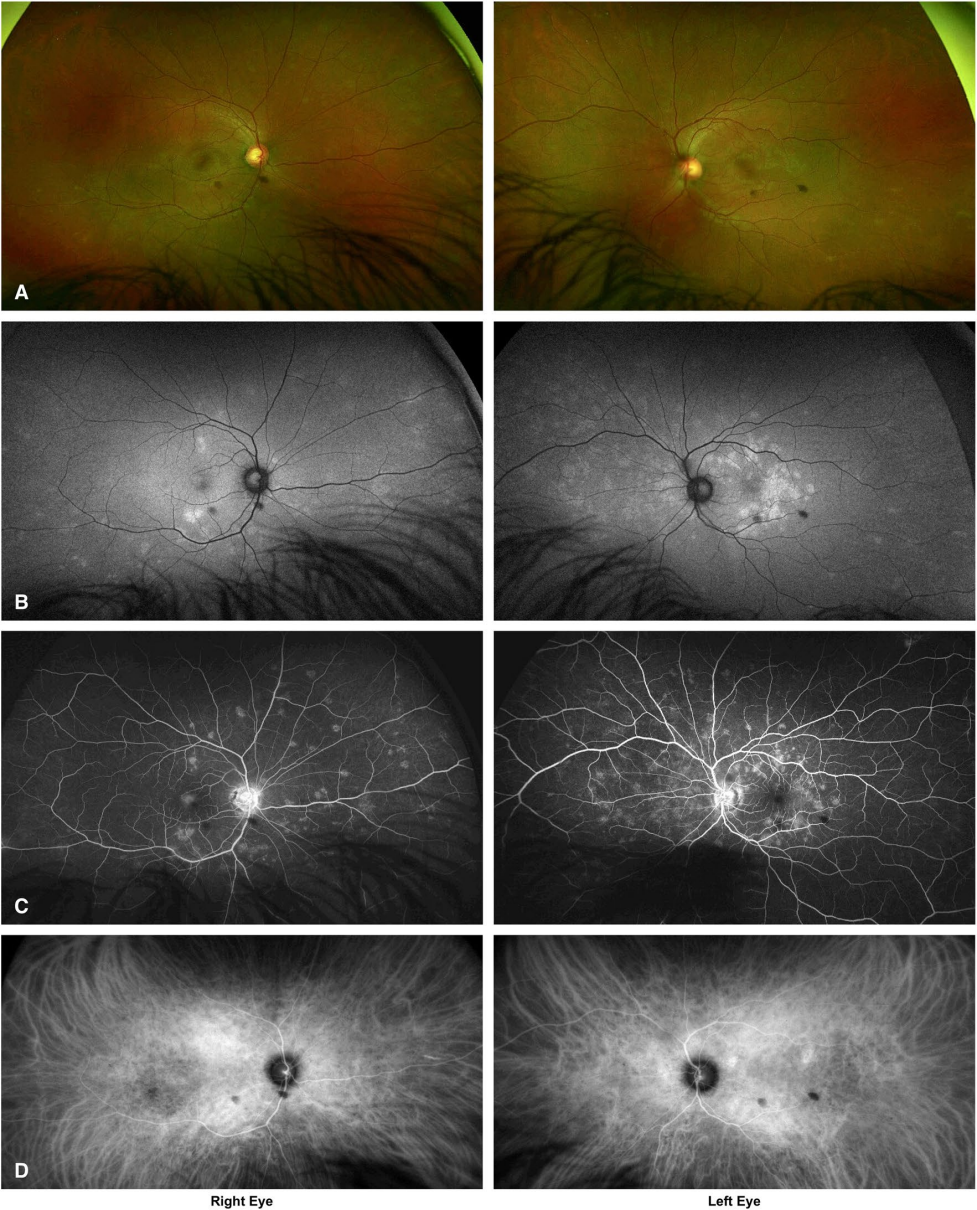
**A**–**D**: Diagnostic imaging at presentation. Ultra-widefield imaging (**A**) shows multiple pale lesions in both eyes. Fundus autofluorescence (**B**) highlights corresponding hyperautofluorescent spots. Fluorescein angiography (**C**) reveals early hyperfluorescent lesions, and indocyanine green angiography (**D**) demonstrates mid-phase hypofluorescent spots, consistent with Multiple Evanescent White Dot Syndrome. The two dark circular spots visible in all images are artifacts likely caused by dust on the Optomap imaging lens

Ultra-widefield imaging revealed multiple small, pale, white retinal lesions in both eyes, corresponding to hyperautofluorescent spots that were more easily detected on FAF (Fig. 1A and B). FA showed wreath-like hyperfluorescent spots in the early phase of the angiogram corresponding to the pale retinal lesions (Fig. 1C). Small, hypofluorescent spots throughout both fundi were visible on ICGA during the middle phase of the angiogram (Fig. 1D).

**Fig. 2:**
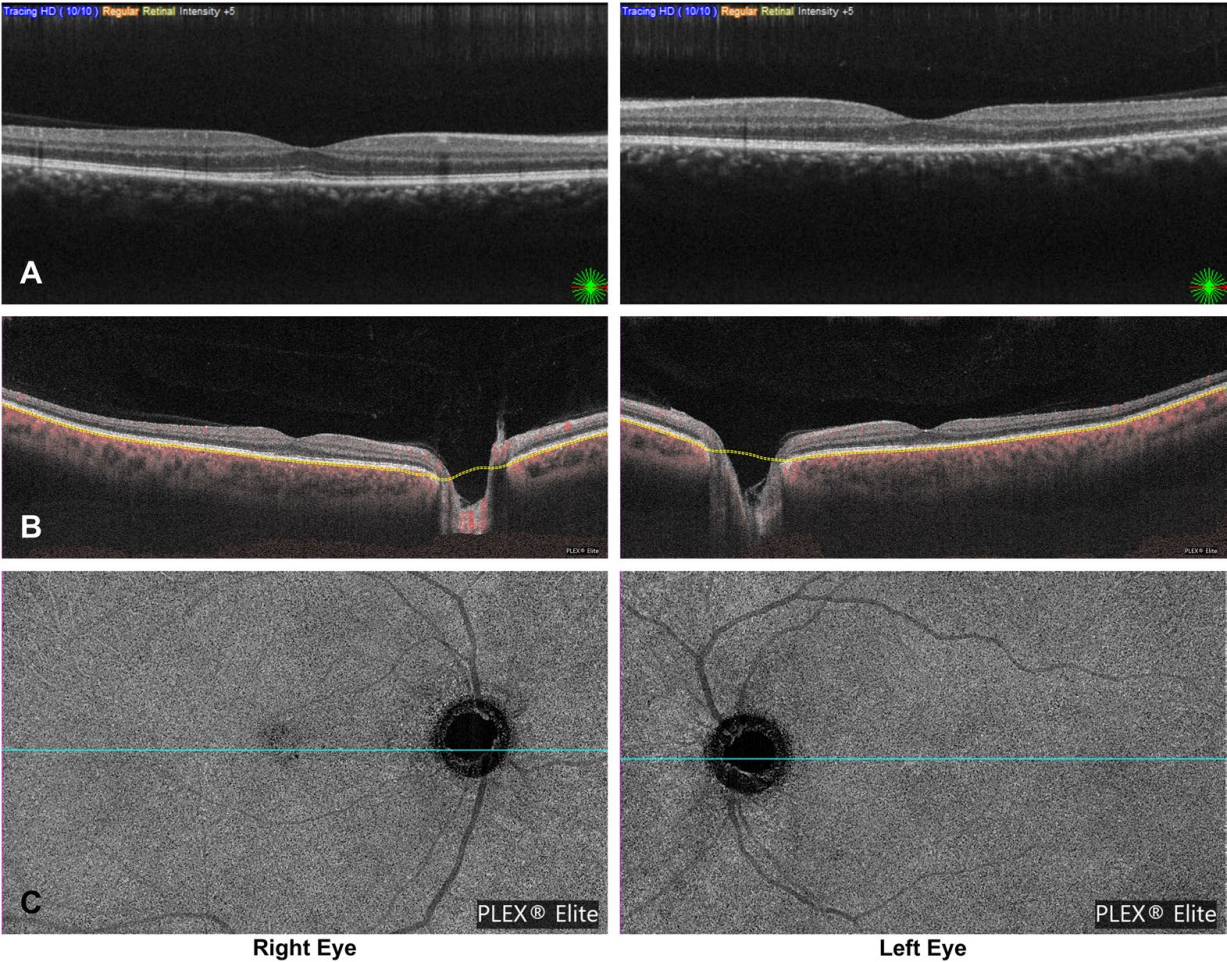
**A**–**C**: Initial multimodal imaging. Spectral-domain OCT (**A**) reveals attenuation of the outer retinal layers. Swept-source OCTA (**B**–**C**) shows a mild subfoveal choriocapillaris flow deficit in the right eye, with no detectable deficit in the left

The SD-OCT showed parafoveal attenuation of the outer retinal layers in the right eye and both foveal and parafoveal attenuation in the left eye (Fig. 2A). The SS-OCTA showed a mild flow deficit in the choriocapillaris in the subfoveal region of the right eye (Fig. 2B and C), while no flow deficit was visible in the left eye (Fig. 2B and C). Based on clinical and imaging findings, a working diagnosis of bilateral MEWDS was established, and no treatment was initiated.

**Fig. 3:**
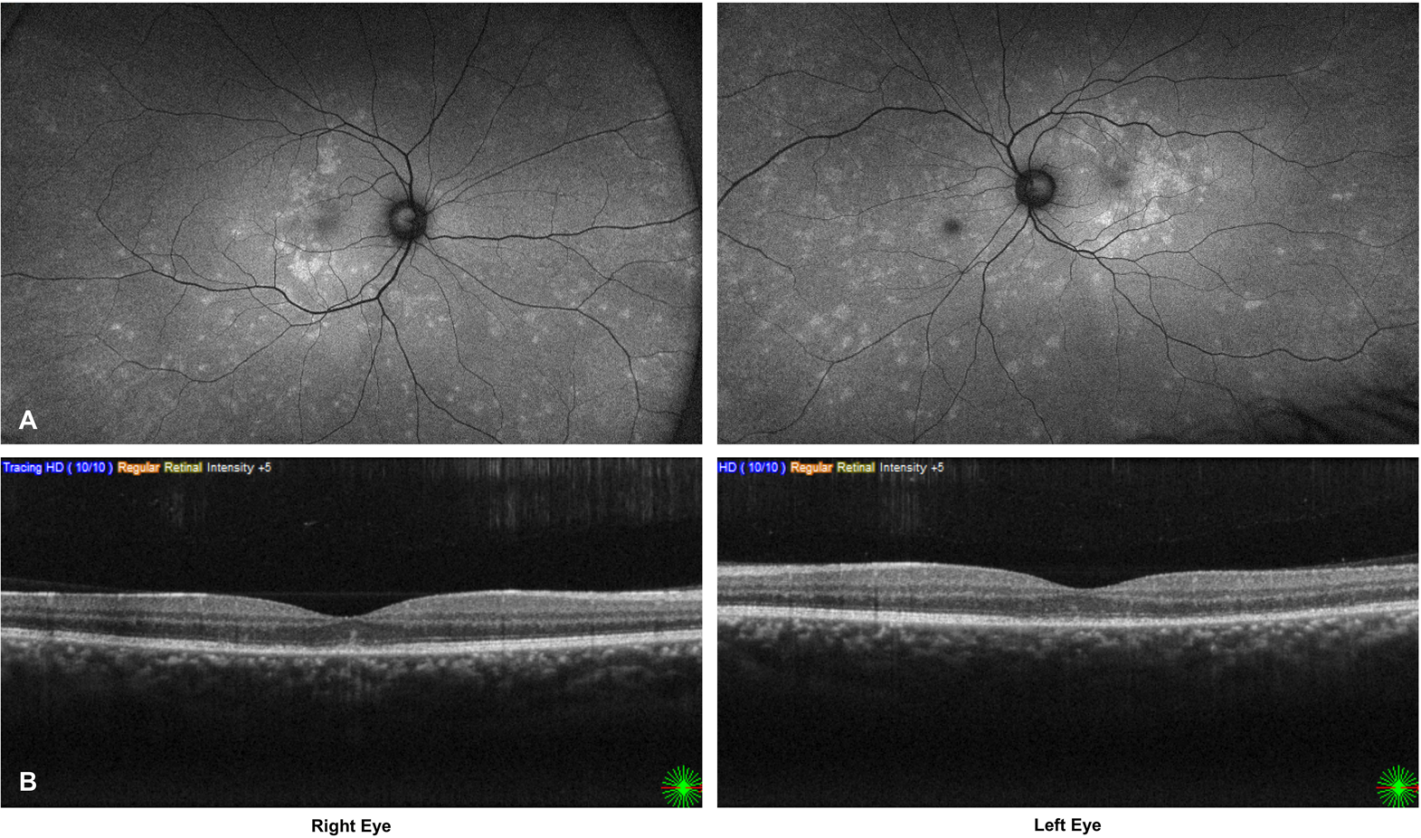
**A**–**B**: Follow-up after two weeks. Fundus autofluorescence (**A**) shows increased macular involvement in the right eye. Spectral-domain OCT (**B**) demonstrates further outer retinal attenuation in the right eye and partial restoration in the left, correlating with functional changes

Approximately two weeks later, the patient reported deterioration of vision in the right eye and slight improvement in the left eye. His best-corrected visual acuity had decreased to 20/40 in the right eye and marginally improved to 20/40 in the left eye. These changes were consistent with FAF and SD-OCT findings, which demonstrated increased macular involvement in the right eye (Fig. 3A and B). In the left eye, SD-OCT showed partial restoration of the outer retinal segments, correlating with the modest improvement in BCVA.

The patient’s scotomas gradually disappeared in both eyes, resolving entirely eight weeks after the first visit. At this time, he again developed a headache and was referred for an MRI, which showed no evidence of cerebral vasculitis.

**Fig. 4:**
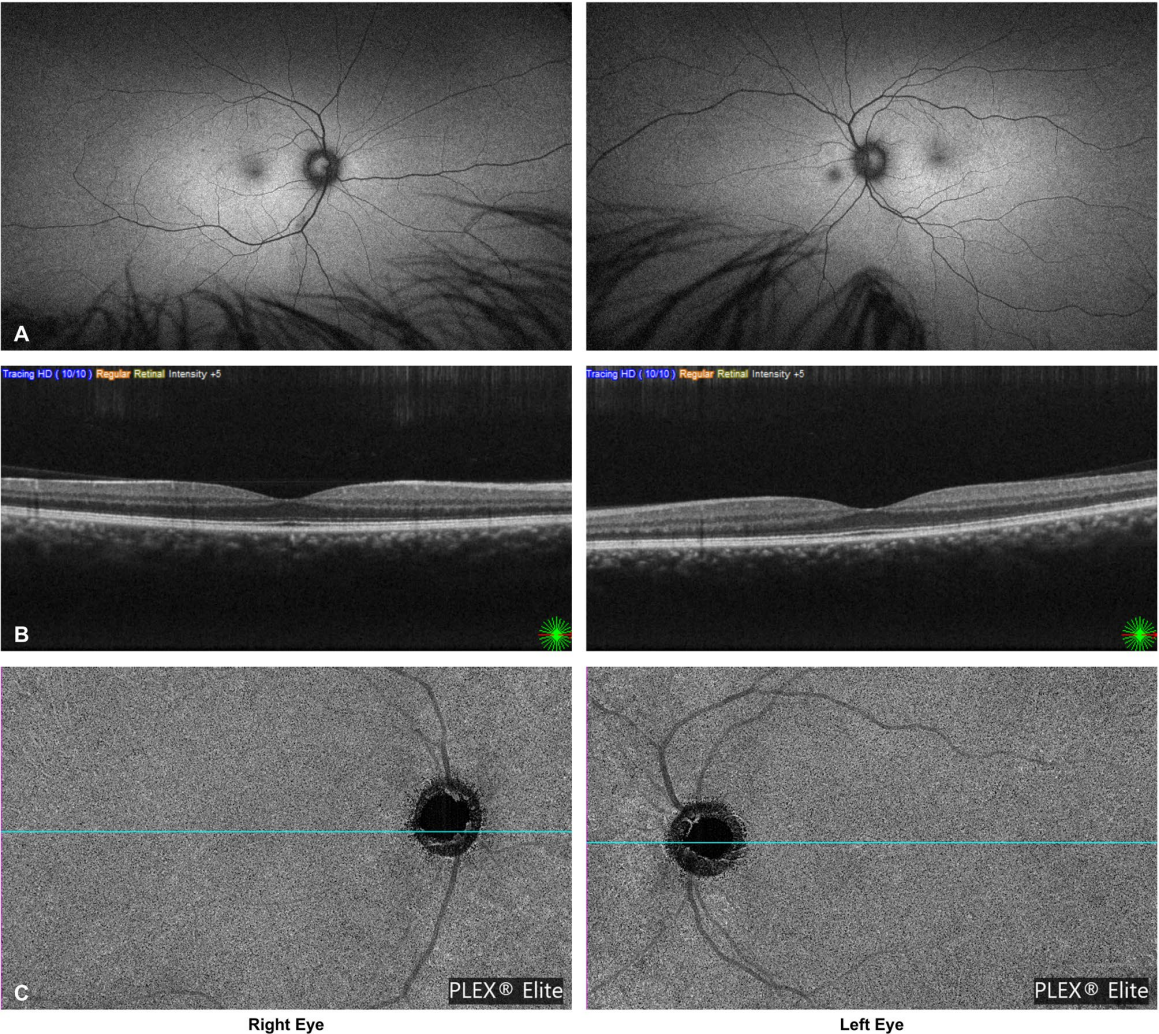
**A**–**C**: Final follow-up at 11 months. Fundus autofluorescence (**A**) and swept-source OCTA (**B**–**C**) show complete resolution, with no residual choriocapillaris flow deficit

Three months after presentation, the patient still reported visual haze in both eyes; however, the BCVA had improved to 20/16 bilaterally. At the final follow-up, 11 months after symptom onset, BCVA was maintained at 20/16 without visual symptoms. Fundus autofluorescence photography and SD-OCT had normalized, and there was no remaining choriocapillaris flow deficit in the right eye on SS-OCTA (Fig. 4A–C).

## Discussion

This case highlights the diagnostic challenge of distinguishing between bilateral MEWDS and APMPPE. Although MEWDS is typically unilateral, bilateral presentations have been reported and may complicate diagnosis. APMPPE has been associated with systemic vasculitis and neurological complications [7], which may necessitate systemic corticosteroid therapy and neurological evaluation [12]. Differentiating between these entities is therefore critical to avoid unnecessary interventions and missed diagnoses.

MEWDS is recognized as a primary choriocapillaritis rather than a photoreceptoritis, with inflammation and hypoperfusion of the choriocapillaris causing secondary, reversible outer retinal damage [3]. One study using SS-OCTA demonstrated flow preservation within the choriocapillaris in MEWDS patients [11]. Other OCTA studies, however, have shown partial or transient choriocapillaris flow deficits in MEWDS that resolve with clinical recovery [4, 13]. The latter studies, including ours, support the pathophysiologic model of transient ischemia at the level of the choriocapillaris.

The Plex Elite 9000 SS-OCTA allowed detailed, serial visualization of choriocapillaris perfusion changes in this case. However, SS-OCTA may fail to detect end-capillary dysfunction due to the intrinsically low flow in these vessels, which may be below the detection threshold, or due to the transient and patchy nature of the ischemia [3]. This technical limitation must be considered when interpreting negative OCTA findings in suspected MEWDS and should not be used as a substitute for multimodal imaging. However, it could have a place in differentiating between these entities.

To our knowledge, this is the first reported case of bilateral MEWDS followed longitudinally with Plex Elite 9000 SS-OCTA, highlighting its utility in documenting both structural and perfusion recovery.

## Data Availability

Not applicable.

## Abbreviations

MEWDS: Multiple Evanescent White Dot Syndrome
APMPPE: Acute Posterior Multifocal Placoid Pigment Epitheliopathy
SS-OCTA: Swept-Source Optical Coherence Tomography Angiography
BCVA: Best-Corrected Visual Acuity
RPE: Retinal Pigment Epithelium
SD-OCT: Spectral-Domain Optical Coherence Tomography
OCTA: Optical Coherence Tomography Angiography
FAF: Fundus Autofluorescence
FA: Fluorescein Angiography
ICGA: Indocyanine Green Angiography
MRI: Magnetic Resonance Imaging
PICCPs: Primary Inflammatory Choriocapillaropathies
